# Increased Expression of Pyroptosis in Leukocytes of Patients with Kawasaki Disease

**DOI:** 10.3390/diagnostics11112035

**Published:** 2021-11-03

**Authors:** Kuang-Che Kuo, Ya-Ling Yang, Mao-Hung Lo, Xin-Yuan Cai, Mindy Ming-Huey Guo, Ho-Chang Kuo, Ying-Hsien Huang

**Affiliations:** 1 Department of Pediatrics, Kaohsiung Chang Gung Memorial Hospital and Chang Gung University College of Medicine, Kaohsiung 833, Taiwan; light@cgmh.org.tw (K.-C.K.); trentlo@cgmh.org.tw (M.-H.L.); l11722@yahoo.com.tw (X.-Y.C.); mindymhguo@cgmh.org.tw (M.M.-H.G.); 2 Kawasaki Disease Center, Kaohsiung Chang Gung Memorial Hospital, Kaohsiung 833, Taiwan; 3 Department of Anesthesiology, Kaohsiung Chang Gung Memorial Hospital, Kaohsiung 833, Taiwan; inr453@cgmh.org.tw

**Keywords:** Kawasaki disease, coronary arterial lesions, pyroptosis, caspases, IVIG resistance

## Abstract

Background: Kawasaki disease (KD) is a form of febrile vasculitis that primarily occurs in children. It can cause inflammation of the coronary arteries, which leads to aneurysms. The pathogenesis of coronary arteries may be associated with apoptosis or pyroptosis mediated by caspases activity, but this idea has not been discussed much in KD. Materials and Methods: We enrolled 236 participants in this study. In the Affymetrix GeneChip^®^ Human Transcriptome Array 2.0 study, there were 18 KD patients analyzed prior to receiving intravenous immunoglobulin (IVIG) treatment, at least 3 weeks after IVIG treatment, and 36 non-KD control subjects. We also recruited 24 KD patients prior to receiving IVIG treatment, at least 3 weeks after IVIG treatment, and 24 non-KD control subjects for Illumina HumanMethylation450 BeadChip study. A separate cohort of 134 subjects was analyzed to validate real-time quantitative PCR. Results: The mRNA levels of caspase-1, -3, -4, and -5 were significantly increased in KD patients compared with control subjects (*p* < 0.05). After administration of IVIG, the expression of these genes decreased considerably. Of particular note, the methylation status of the CpG sites of the caspase-4 and -5 genes demonstrated significant opposite tendencies between the KD patients and controls. Furthermore, compared with patients who responded to IVIG, refractory KD patients had a lower expression of the caspase-3 gene prior to IVIG treatment. Conclusion: Our study is the first to report the upregulation of pyroptotic caspase-1, -4, and -5 in peripheral leukocytes of KD patients. Moreover, the expression of caspase-3 may be associated with IVIG resistance in KD.

## 1. Introduction

Kawasaki disease (KD) is a form of febrile system vasculitis that mostly affects children under the age of 5 years old [1]. Although the pathogenesis of KD is still unknown, it has tentatively been defined as having an infection-immuno-genetic pathogenesis [2]. A single 2 mg/Kg dosage of intravenous immunoglobulin (IVIG) is currently the main treatment for coronary artery lesions (CAL) for KD patients [3]. Global studies have also provided rational strategies for exploring the optimal treatment of this disease [4,5,6,7]. Furthermore, the effect of Casp-1 and NLRP3 inflammasome on the endothelial cell pyroptosis of KD has been previously studied by in vitro models [8,9]. Stress-mediated caspase-3 activation in endothelial progenitor cells might be involved in hyper-homocysteinemia-induced coronary heart disease [10]. Protective effects have been reported in Casp-1-, NLRP3-, IL-1a-, IL-1b-, and IL-1R-deficient mice challenged with *Lactobacillus casei* cell wall extract (LCWE) [11]. Recently, the human IL-1 receptor antagonist, anakinra, has been in a clinical trials for treating acute KD [12].

High-dose IVIG can modulate and suppress the human inflammatory-immune system. Furthermore, recent studies have shown that the epigenetic hypomethylation of individual mRNA by IVIG can be used to partially explain the pathogenesis of KD [7,13,14,15,16,17]. Toll-like receptors (TLRs) are the sensors of the innate immune system and they can induce proinflammatory cytokine expressions [18]. Recently, researchers have strongly suggested that KD is also an autoinflammatory-like disease and is characterized by the activation of inflammasomes [8,19]. The caspase network related to inflammasome activation and sequential triggers also plays a key role in the crosstalk between pyroptosis and apoptosis in auto-inflammatory diseases [20]. Our previous study indicated that a microbial inflammatory response may trigger KD via TLRs (especially TLR1, 2, 4, 6, 8, and 9) that induce immune-pathogenesis [13]. Furthermore, NLRs are the intracellular sensors for exogenous pathogenic microbes or endogenous damage-associated molecular patterns [21]. Once NLRs activate inflammasomes, the sequential triggers of the caspase family are recruited to induce the expressions of such proinflammatory cytokines such as IL-1β and IL-18 [8]. In general, certain pathways can activate inflammatory caspases to drive pyroptosis [22]. We also found that IVIG was associated with epigenetic hypomethylation and the upregulation of NOD-like receptors (NLRs) as well as the activation of their downstream interleukin (IL)-1β in KD patients [15].

Epigenetics reveal that the promoter methylation or acetylation of a genome subsequently results in changes in gene expressions between healthy and diseased individuals [23,24]. In the past, several studies have indicated that administration with IVIG altered promoter methylation patterns in KD patients [7,16,25]. As of now, no studies have investigated the gene expressions and methylation profiles in the caspase family of KD patients, although some studies have revealed ethnic CASP3/ITPKC polymorphisms, and risks for IVIG unresponsiveness and CAL formation [26,27,28]. Therefore, we performed a comprehensive examination of the mRNA expressions of these caspases family and analyzed the methylation level changes in the KD patients and control subjects.

## 2. Materials and Methods

### 2.1. Patients

First, we enrolled KD patients that met the KD diagnostic criteria of the American Heart Association and who were administered a single dosage of IVIG treatment (2 g/kg) in our hospital. Patients were defined as having IVIG resistance if they remained febrile 48 h after the initial IVIG infusion was completed. In this case–control study, we adopted Affymetrix GeneChip^®^ Human Transcriptome Array 2.0 to compare and quantify the genetic expressions in 18 KD patients (both before and at least 3 weeks after IVIG treatment) as well as in 18 febrile and 18 healthy controls. Furthermore, we used Illumina Human Methylation 450 BeadChip to study the promoter methylation in 24 KD patients prior to undergoing intravenous IVIG treatment and at least 3 weeks after IVIG treatment as well as in 12 febrile and 12 healthy controls.

We further validated the target genes in a separate cohort of 46 KD patients, 44 febrile subjects, and 44 healthy subjects using real-time quantitative PCR. The patients in the fever control group had been diagnosed with acute pharyngitis, tonsillitis, bronchopneumonia, or urinary tract infection. The peripheral blood samples were collected from KD patients both before receiving IVIG treatment (pre-IVIG) and after completing IVIG treatment [16]. CAL was defined through echocardiography as the severity of the coronary being classified using Z scores according to the 2017 AHA statement [29,30] According to a protocol at our hospital, the patients received echocardiography at the following time points: at the time of diagnoses and one week, one month, two months, and six months after diagnosis. This study was approved by the Chang Gung Memorial Hospital Institutional Review Board, and we obtained written informed consent from the parents or guardians of all participants. The subjects were permitted to withdraw from the study at any time during the study period.

### 2.2. Experiment Design

We first collected whole blood samples from the subjects and submitted them to WBC enrichment, as previously described in another study [13]. The enriched WBC samples were then subjected to either RNA or DNA extraction.

#### 2.2.1. DNA Methylation Profiling with Illumina M450K BeadChip

We adopted Illumina HumanMethylation450 (M450K) BeadChip to study the DNA methylation patterns as previous described [31]. M450K BeadChip was designed to detect methylation patterns of approximately 450,000 CpG markers that span the human genome. Briefly, we used 200 ng of bisulfite-converted genomic DNA in accordance with the manufacturer’s instructions. We then calculated the methylation percentage of cytosine for each CpG marker in each sample, which we referred to as the β value. We analyzed the generated raw data using Partek. All DNA methylation data were submitted to NCBI GEO; please refer to GSE109430 for further information. More information about M450 BeadChip can be found at http://support.illumina.com/array/array_kits/infinium_humanmethylation450_beadchip_kit.html (accessed on 19 January 2018).

#### 2.2.2. Gene Expression Profiling with Microarray

To obtain unbiased results, we created pooled RNA libraries by evenly pooling six RNA samples, which resulted in three pooled pre-IVIG and three post-IVIG libraries of KD as well as three healthy control and three fever control pools, as previously described [7]. We performed a microarray assay on the pooled RNA samples to establish the gene expression profiles and then profiling with GeneChip^®^ Human Transcriptome Array 2.0 (HTA 2.0, Affymetrix, Santa Clara, CA, USA). We measured the RNA concentrations with the NanoDrop 2000 spectrophotometer (Thermo Scientific, Waltham, MA, USA). All RNA samples passed the criterion of a RIN ≥ 7 when assessed using the Agilent 2100 Bioanalyzer (Agilent, CA, USA), as described in a previous study [31]. We prepared the RNA samples with the WT PLUS Reagent kit and performed hybridization on the HTA 2.0 microarray chips. We subjected the HTA 2.0 chips’ raw data to quality control examination and then analyzed the data with Partek [31]. The microarray data were referred to NCBI GEO; (GSE109351) [31].

#### 2.2.3. RNA Isolation and Real-Time Quantitative RT-PCR

To quantify the mRNA levels of CASP1, CASP3, CASP4, and CASP5, we adopted the LightCycler^®^ 480 Real-Time PCR System (Roche Diagnostics International AG Rotkreuz, Switzerland) to perform real-time quantitative PCR. The housekeeping gene of 18S was used for the internal control. We separated the total mRNA from the peripheral white blood cell (WBC) by using an isolation kit (mirVana™ miRNA Isolation Kit, Catalog number: AM1560, Life Technologies, Carlsbad, CA, USA) and then calculated both the quality (RIN value) and quantity of the RNA samples with Bioanalyzer (ABI) and Qubit (Thermo), respectively, in accordance with the manufacturer’s instructions. We performed PCR using a SYBR Green PCR Master Mix containing 10 μM of specific forward and reverse primers. We performed the relative quantification of gene expression based on the comparative threshold cycle (C_T_) method, which enabled us to determine the target amount as 2^−(ΔCT target − Δ CT calibrator)^ or 2^−ΔΔCT^. We designed the primers to amplify the target genes, as shown in Table 1. We performed all experiments twice in order to verify and validate the amplification efficiencies.

#### 2.2.4. Functional Study to Validate the Expression of CASP 1/4/5 in KD Patients

To further study the mechanisms, a monocyte cell line (U937) treated with plasma of KD or healthy control was applied to investigate the expressions of CASP1, 4, and 5 in regulating KD in vitro. The human macrophage cell line U937 cells (ATCC and CRL-1593) were maintained in a RPMI1640 cell culture medium (Thermo Fisher Scientific) with 10% FBS at 37 °C, 5% CO_2_. U937 monocytes were differentiated to macrophage cells by adding phorbol 12-mystrate 13-acetate (PMA, Thermo Fisher Scientific) for 48 h [32]. After differentiation, the cells were plated at a density of 2 × 10^5^ cells/well in 12-well plates and cultured in RPMI1640 medium supplemented with 10% *(vol/vol)* pooled healthy control plasma or pooled KD plasma from 10 individuals for 72 h; then, the cells were collected, and the RNA were extracted. We used three independent experiments for this study (N = 30 of KD patients and N = 30 of healthy controls). Real-time PCR was performed to quantify the mRNA levels of CASP1, CASP4, and CASP5.

### 2.3. Statistical Analysis

All data are presented as mean ± standard error. Once the chips met the quality control criteria, they were evaluated using Partek (Partek, St. Louis, MO, USA), which is commercial software specifically designed to analyze microarray data. For setting up the correlation of transcription (HTA2.0) and methylation (M450K) microarray, we performed 10,000 re-samplings for each genes to extract pairs of gene intensity and CpG marker β values in each group. Therefore, we collected 40,000 pairs of gene intensity and CpG marker β values from the four groups [12]. We adopted one-way ANOVA or Student’s *t*-test, as appropriate, to evaluate the quantitative data and paired sample *t*-test to evaluate any data changes before and after IVIG treatment. We carried out all statistical analyses with SPSS version 12.0 for Windows XP (SPSS, Inc., Chicago, IL, USA), and a two-sided *p*-value less than 0.05 was considered statistically significant.

## 3. Results

### 3.1. Promoter Hypomethylation and Upregulation of Caspases mRNA Levels in KD Patients

The lower a CpG marker is methylated, the more abundantly the gene is expressed [17]. In the beginning, we chose the ideal genes for possessing a negative correlation with CpG markers with a 5% change in M450K and a two-fold change in HTA 2.0 between the KD patients and controls. Then, we selected a single site of CpG methylation for the correlation analysis according to a significant *p*-value and the largest difference [25].

**Figure 1:**
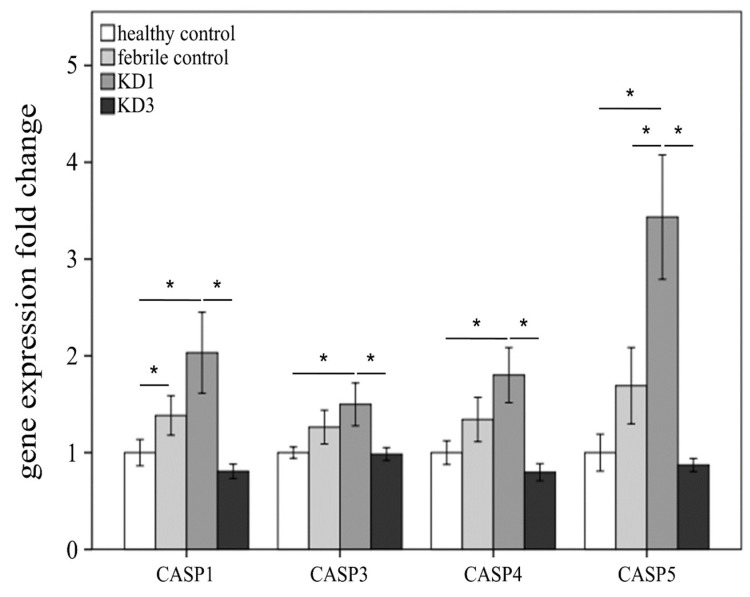
Analyses of caspase mRNA expressions using GeneChip^®^ Human Transcriptome Array 2.0 (HTA 2.0) between Kawasaki disease (KD) patients and control subjects. The chips that passed the quality control criteria were analyzed with Partek (Partek, St. Louis, MO, USA), a commercial software specifically for microarray data analysis. Using Partek, we conducted ANOVA analysis and reported the *p*-value of the comparisons of interest. KD1: Kawasaki disease before IVIG treatment; KD3: Kawasaki disease > 3 weeks after IVIG treatment; FC: febrile control; HC: healthy control. Data are expressed as mean ± standard error. * indicates significance (*p* value < 0.05).

**Figure 2:**
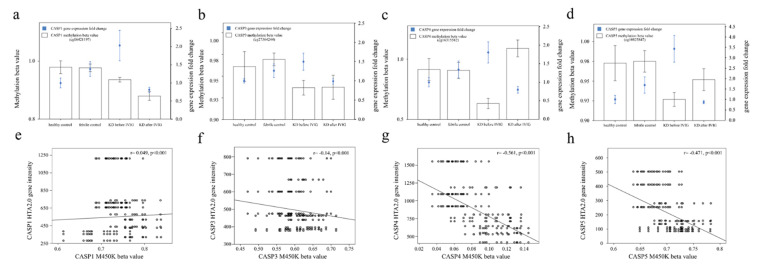
Integration of DNA methylation (M450K) and gene expression (HTA 2.0) profiles of CASP1 (**a**), CASP3 (**b**), CASP4 (**c**), and CASP5 (**d**). We used scatter plots (**e**–**h**) to represent the relationship between mRNA and rgw methylation levels of the CpG markers. The mRNA expression levels and cg16315582 of CASP4 (**g**) and cg10825847 of CASP5 (**h**) have a negative correlation in Kawasaki disease (KD) and control subjects (Pearson’s correlation coefficient r = −0.561 and −0.471, all *p* < 0.001). The histogram and curve are presented as mean ± standard error. We performed 10,000 re-samplings for each genes to extract pairs of gene intensity and CpG marker β values in each group. Therefore, we collected 40,000 pairs of gene intensity and CpG marker β values from the four groups. Using Partek, we conducted an ANOVA analysis and reported the *p*-values of the comparisons of interest. KD1: Kawasaki disease before IVIG treatment; KD3: Kawasaki disease > 3 weeks after IVIG treatment; FC: febrile control; HC: healthy control.

Initially, we studied the variations in epigenetic and genetic profiles of caspases between the controls and KD patients before and after IVIG treatment. First, we investigated the 14 genes of the caspase family (Table 2). The expression of these caspase genes were compared in four groups in the transcription levels of the HTA2.0 microarray. Of particular note, CASP1, CASP3, CASP4, and CASP5 were, respectively, significantly upregulated in KD patients compared with in the febrile or healthy controls and significantly decreased following IVIG administration. Therefore, the gene expressions of CASP1, CASP3, CASP4, and CASP5 were implied to lead a trend of KD and the regulation of CASP1/5 could play an important role (Figure 1). In Figure 2, we integrated the DNA methylation and gene expressions of CASP1, CASP3, CASP4, and CASP5 compared in four groups. The trend of higher expression combined with lower DNA methylation of these genes was noted in acute KD. Furthermore, the promoter methylation and mRNA expression levels of CASP4 (cg16315582) and CASP5 (cg10825847) have a significant negative correlation between KD and control subjects (Pearson’s correlation coefficient r = −0.561 and −0.471, all *p* < 0.001).

### 3.2. Increased CASP1, CASP3, CASP4, and CASP5 Expressions in the WBC of KD Patients

**Figure 3:**
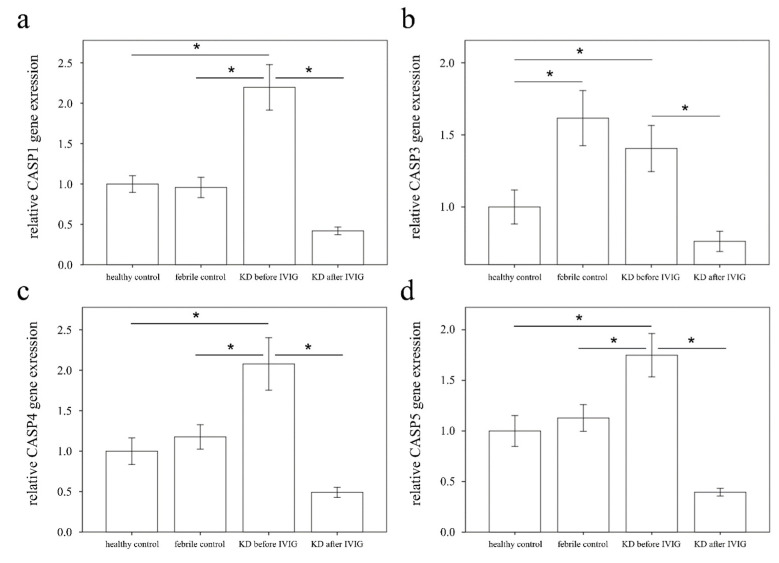
Analyses of CASP1 (**a**), CASP3 (**b**), CASP4 (**c**), and CASP5 (**d**) in the peripheral white blood cells of 46 Kawasaki disease (KD) patients before and after intravenous immunoglobin treatment (IVIG) as well as 44 healthy and 44 febrile controls using a real-time quantitative polymerase chain reaction. Data are expressed as mean ± standard error. Data analyzed by one way ANOVA and Fisher’s LSD multiple comparisons. * indicates significance (*p* < 0.05) between the groups.

**Figure 4:**
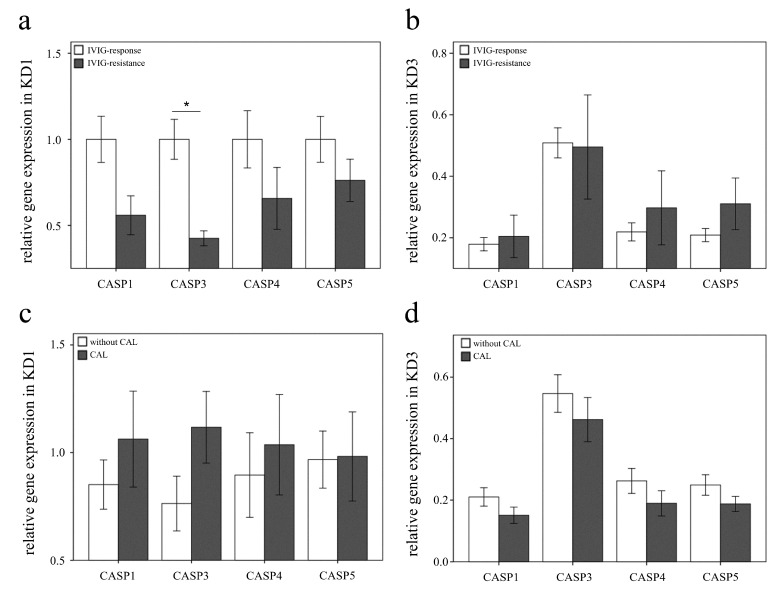
The relationship of the expressions of CASP1, CASP3, CASP4, and CASP5 analyzed in intravenous immunoglobin (IVIG) response (**a**,**b**) and coronary artery lesions (CAL) formation (**c**,**d**) respectively in peripheral leukocytes of 46 Kawasaki disease (KD) patients, using a real-time quantitative polymerase chain reaction. Data analyzed by one way ANOVA and Fisher’s LSD multiple comparisons. * indicates significance (*p* < 0.05) between the groups. KD1: Kawasaki disease before IVIG treatment; KD3: Kawasaki disease > 3 weeks after IVIG treatment.

We subsequently executed qPCR assays to investigate the mRNA levels of CASP1, CASP3, CASP4, and CASP 5 in a separate cohort of 46 KD patients, 44 febrile controls, and 44 healthy controls (Table 3). As a result, we found elevated CASP1, 4, and 5 in KD patients compared with those of the febrile and healthy controls, and the CASP1, 3, 4, and 5 levels considerably decreased 3 days after IVIG administration (all *p* < 0.001), as shown in Figure 3. These results are in line with HTA 2.0. Interestingly, Casp-1, -4, and -5 participate in both canonical and non-canonical inflammasome-related pyroptosis. The expression of CASP3 in KD patients is significantly higher than in healthy controls but similar to febrile controls. We further found significant differences in the CASP3 mRNA levels between KD patients with resistance to IVIG treatment (0.4 ± 0.04) and those without (1.0 ± 0.12) prior to IVIG administration (*p* < 0.001) (Figure 4). However, following IVIG administration, the expression of CASP3 was conversely upgraded in the IVIG-resistant group compared with its downregulation in the group of IVIG responsiveness. The CASP3 expressions of CAL +/− were, respectively, 1.1 ± 0.17 and 0.8 ± 0.13, which was not different significantly (*p* = 0.096).

### 3.3. The Increased Expression of CASP5 in U937 Cells Stimulated with Plasma of KD

**Figure 5:**
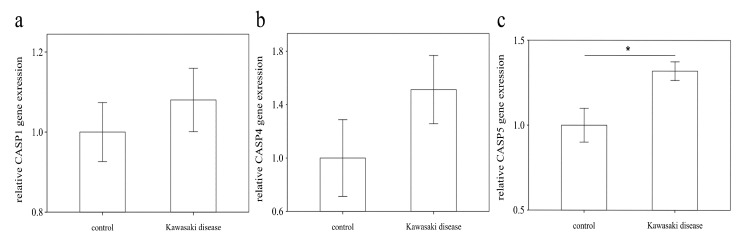
Analyses of CASP1 (**a**), CASP4 (**b**), and CASP5 (**c**) mRNA levels in human monocyte cell (U937) experiments after treatment of 10% of pooled KD patients’ plasma and pooled healthy control from 10 individuals for 72 h in vitro, using a real-time quantitative polymerase chain reaction. Data are expressed as mean ± standard error. Data analyzed by Student’s *t*-test and reported as the *p*-values of the comparisons of interest. * indicates significance (*p* < 0.05) between the groups. KD: Kawasaki disease.

In this vitro study (Figure 5), the expression of CASP5 was significantly higher in KD plasma treatment than in the healthy control plasma treatment (*p* < 0.05). The mRNA levels of CASP1 and CASP4 were upregulated in the KD group compared with in the healthy control but were not statistically significant. This work may indicate that CAPS5 could play a more important role in pyroptosis in human macrophages of KD.

## 4. Discussion

Inflammatory caspases (caspase-1, -4, and -5) are vital for human innate immune defenses, and this study is the first in which a comprehensive survey of transcripts of 14 caspases is performed in KD patients. Our major findings include that KD patients have a trend of higher expressions of caspase-1, -3, -4, and -5 when compared with the control subjects. Our finding suggests that inflammasome pathways of pyroptosis in peripheral leukocytes may play an important role in the inflammatory pathogenesis of KD. The expression of caspase-3 in KD patients is significantly higher than in healthy controls but similar to febrile controls. We further found significant differences in the CASP3 mRNA levels between KD patients with resistance to IVIG treatment and those without prior to IVIG administration.

Inflammatory caspases 1, 4 and 5 are activated in response to microbial infection such as SARS-CoV-2 and danger stress and then trigger pyroptosis [33,34,35,36,37,38]. Inflammatory caspases are formed as an inactive monomer. Once activated, they are recruited into multi-protein complexes known as inflammasomes [39] and they cleave gasdermin D to generate an N-terminal cleavage product for executing pyroptosis and releasing IL-1ß [40,41,42]. Our previous work [15] and this study (Table 2) showed that the expressions of mRNA levels of NLRP3, IL-1a, ASC, and GSDMD were not significantly upregulated in peripheral WBCs of patients with KD compared with the controls. Moreover, our previous studies showed that pyroptosis of TLR stimulation, inflammasome complex activation (NLRC4 and NLRP12), and sequential IL-1ß and IL-18 upregulation were all associated with the inflammatory pathways of KD [13,15,43]. This finding suggests that both canonical (capase-1) and non-canonical (caspase 4/5) pathways of pyroptosis in peripheral leukocytes may play important roles in the inflammatory pathogenesis of KD. Wang WT et al. showed similar data on caspases-1, -4, and -5 in the peripheral leukocytes of KD patient and MIS-C (Multisystem Inflammatory Syndrome in Children) [44]. They also indicated that the caspase-4/5-dependent noncanonical inflammasome in granulocytes was more unique to MIS-C with small cases. The importance of caspase 1 in KD has been established in the LCWE and CAWS model of Kawasaki Disease vasculitis. It is an interesting finding that caspase 4 and 5 may also contribute to disease pathogenesis. Furthermore, we found that the activation of caspase-5 may play an important role in the inflammatory process of macrophages/monocytes in KD patients (Figure 5). Consistently, recent studies indicate that pyroptosis induced by Gram-negative bacteria is not initiated by a traditional inflammasome but merely through a single caspase that acts as both the receptor and pyroptotic initiator such as caspase4/5 or 11 [45,46]. Therefore, this finding may entice researchers to conduct a study of caspase 11 in one murine model of KD in the future.

A single high dosage of IVIG is currently the main treatment for CAL complication of KD. In 2016, a study by Li et al. indicated that IVIG can significantly alter methylation on the promoters of inflammatory-associated genes in KD [25]. Likewise, this study demonstrated that a single high dose of IVIG results in the downregulation of the expression of caspase-4 and -5 genes regarding DNA methylation. Of the 46 KD cases in this study of the RT-qPCR assay, 22 (47.8%) cases had CAL complications. However, regarding IVIG treatment for the expression of the caspase-1, -3, -4, and -5 genes, we observed no significant differences between KD patients with and without CAL. This result indicates that such a caspase activation in peripheral leukocytes may not significantly affect the mechanism of the consequent formation of CAL, which was not in line with previous studies on endothelial cells [8,11]. Moreover, the expression of CASP3 is lower significantly with IVIG resistance (Figure 4a). Although the expression CASP3 regarding CAL formation is higher, it is not significant statistically (Figure 4c). The results of this study might indicate that the expression of CASP3 in peripheral leukocytes play a role of IVIG resistance but not of CAL formation. Further analysis and study may be conducted to understanding this mechanism in the future.

The upregulation of caspase-3 gene expression differs significantly in KD patients before IVIG administration compared with the control subjects. Interestingly, caspase-3 also seems to play a role regarding IVIG resistance in KD patients. Inflammasomes activate a network of caspases, such as caspase-1,-3, and -8, and can promote both pyroptotic and apoptotic cell death [47]. Caspase-3 is the main enzyme that executes programmed cell death when receiving signals from both extrinsic (caspase-8 and 10) and intrinsic (caspase 9) pathways of apoptosis. As shown in Figure 4, caspase-3 expression in the IVIG resistance group is significantly lower than the group of IVIG responsiveness prior to IVIG treatment. Except for caspase-3, the expression values of all caspases decreased in both groups following IVIG administration. Such IVIG non-responders were found to be slow in the regulation of caspase-3 gene expression by IVIG therapy (Figure 4). This phenomenon may be the result of individual genetic differences or other inflammation mechanisms [48]. Polymorphic variants of caspase-3 and the caspase-3 activation have been found to predict the risk of CAL with regard to apoptotic pathways [15,48,49]. Kuo et al. also found that Taiwanese SNPs in the A allele of CASP3(rs72689236) are more frequently found in IVIG resistance and CAL formation in KD [27]. A study from Japan reported that G to A substitutions of a single-nucleotide polymorphism (SNP) located in CASP3 (rs72689236)/ITPKC, which was associated with nuclear factor of activated T cell-mediated T-cell activation, is responsible for susceptibility and IVIG resistance to KD [28]. Moreover, Onouchi et al. also revealed that the A allele of CASP3 (rs72689236) more frequently resulted in lower caspase-3 mRNA expression in KD patients. This finding was in line with our finding about lower caspase-3 expression in IVIG resistance than IVIG responsiveness in KD. We supposed that it might be associated with the variant SNPs of A allele frequency. Furthermore, a clinical trial of cyclosporine as supplemental therapy for refractory KD was recently conducted in Japan regarding variant SNPs of CASP3/ITPKC [5]. Therefore, further studies are needed to better understand the role of caspase-3 in early diagnostic biomarkers of blood or supplemental therapy of refractory KD patients in Taiwan.

This study has certain limitations. As we used peripheral leukocytes for the study of each subject, the expression of caspase genes from single cells such as monocyte or neutrophil was not distinguished in this study. Furthermore, since the amount of each blood sample from infants or children was small, a translation performance of caspase genes by Western blot analysis was not performed in this study.

## 5. Conclusions

Insight into the mechanisms that control caspase activation in KD will help us better understand the immmopathogenesis of KD. Our study is the first to observe DNA hypomethylation and increased caspase transcripts of peripheral leukocytes in KD. This study suggests that the upregulation of pyroptotic caspases (−1, −4, and −5) plays an important role in the inflammatory pathways of peripheral WBCs in KD. Caspase-3 expression is associated with response to IVIG therapy in KD, and its expression can be a biomarker of refractory KD in the Taiwanese population. However, further research is still warranted in the future.

## Figures and Tables

**Table 1:** Primers for genes.

Gene Symbol	Accession Number	Hybridization	Primers (5′ to 3′)
RNA18S5	NR_003286.2	forward	GTAACCCGTTGAACCCCATT
		reverse	CCATCCAATCGGTAGTAGCG
CASP1	NM_001223	forward reverse	TTTCCGCAAGGTTCGATTTTCA GGCATCTGCGCTCTACCATC
CASP3	NM_004346	forward	AGCGAATCAATGGACTCTGGA
		reverse	GGTTTGCTGCATCGACATCT
CASP4	NM_001225	forward	CAAGAGAAGCAACGTATGGCA
		reverse	AGGCAGATGGTCAAACTCTGTA
CASP5	NM_001136112	forward	ATGGCATCCTAGAGGGAATCT
		reverse	ATGGCATCCTAGAGGGAATCT

**Table 2:** Transcript expressions of caspases (CASPs) between Kawasaki disease patients and control subjects.

Symbol	RefSeq	Column ID	Fold-Change (KD1 vs. HC)	*p* Value (KD1 vs. HC)	Fold-Change (KD1 vs. FC)	*p* Value (KD1 vs. FC)	Fold-Change (KD3 vs. KD1)	*p* Value (KD3 vs. KD1)
CASP1	NM_001223	TC11003505.hg.1	1.993	0.011 *	1.445	0.118	−2.443	0.003 *
CASP2	NM_032982	TC07000932.hg.1	1.172	0.058	−1.026	0.725	−1.303	0.006 *
CASP3	NM_004346	TC04001807.hg.1	1.471	0.035 *	1.182	0.306	−1.495	0.030 *
CASP4	NM_001225	TC11002246.hg.1	1.785	0.019 *	1.346	0.169	−2.235	0.003 *
CASP5	NM_001136112	TC11002247.hg.1	3.417	0.001 *	2.053	0.022 *	−3.811	0.001 *
CASP6	NM_001226	TC04001464.hg.1	1.076	0.119	−1.104	0.046 *	−1.175	0.005 *
CASP7	NM_001227	TC10000825.hg.1	−1.002	0.890	−1.039	0.062	−1.047	0.030 *
CASP8	NM_001080124	TC02001177.hg.1	1.091	0.371	1.047	0.630	−1.039	0.685
CASP8AP2	NM_001137667	TC06000800.hg.1	−1.102	0.565	1.002	0.990	−1.069	0.691
CASP9	NM_001229	TC01002245.hg.1	1.014	0.794	−1.055	0.321	−1.039	0.469
CASP10	NM_032976	TC02001176.hg.1	1.369	0.032 *	−1.010	0.935	−1.535	0.008 *
CASP12	NM_001191016	TC11002244.hg.1	1.016	0.796	−1.058	0.366	−1.005	0.935
CASP14	NM_012114	TC19000266.hg.1	−1.058	0.619	−1.146	0.250	1.066	0.577
CASP16	ENST00000428155	TC16000103.hg.1	−1.033	0.792	−1.070	0.585	1.118	0.378
PYCARD	NM_013258	TC16001052.hg.1	1.183	0.160	−1.033	0.774	−1.174	0.179
IL-1α	NM_000575	TC02002218.hg.1	−1.080	0.335	1.030	0.706	1.067	0.411
GSDMD	NM_001166237	TC08000824.hg.1	1.101	0.153	−1.022	0.726	−1.086	0.214
NLRP3	NM_001079821	TC01002008.hg.1	−1.040	0.750	1.131	0.325	−1.046	0.715
IL-1β	NM_000576	TC02002219.hg.1	1.677	0.066	2.195	0.012 *	−2.475	0.006 *
IL-18	NM_001562	TC11002293.hg.1	1.411	0.012 *	1.115	0.338	−1.417	0.011 *

KD1: Kawasaki disease before IVIG treatment; KD3: Kawasaki disease > 3 weeks after IVIG treatment; FC: febrile control; HC: healthy control. * statistical significance (*p* < 0.05).

**Table 3:** Basal characteristics of patients with KD and controls.

Characteristic	Healthy Controls (n = 44)	Febrile Controls (n = 44)	Patients with KD (n = 46)
Male gender, n (%)	29 (65.9)	23 (53.5)	35 (76.1)
Mean (SD), age (y)	6.0 ± 4.0	3.4 ± 2.6	1.6 ± 1.5
Age range (y)	0–16	0–16	0–9
CAL formation			22 (47.8%)
IVIG resistance			5 (10.9%)

CAL, coronary artery lesion; IVIG, intravenous immunoglobulin; KD, Kawasaki disease.

## Data Availability

All DNA methylation data were submitted to NCBI GEO; please refer to GSE109430 for further information. The microarray data were from NCBI GEO; (GSE109351). More information about M450 BeadChip can be found at http://support.illumina.com/array/array_kits/infinium_humanmethylation450_beadchip_kit.html (accessed on 19 January 2018).
